# Potential Role of Innate Lymphoid Cells in the Pathogenesis and Treatment of Skin Diseases

**DOI:** 10.3390/jcm12083043

**Published:** 2023-04-21

**Authors:** Francesco Borgia, Federica Li Pomi, Clara Alessandrello, Mario Vaccaro, Sebastiano Gangemi

**Affiliations:** 1 Department of Clinical and Experimental Medicine, Section of Dermatology, University of Messina, 98125 Messina, Italy; federicalipomi@hotmail.it (F.L.P.); mario.vaccaro@unime.it (M.V.); 2 Department of Clinical and Experimental Medicine, School and Operative Unit of Allergy and Clinical Immunology, University of Messina, 98125 Messina, Italy; clara.alessandrello@outlook.it (C.A.); sebastiano.gangemi@unime.it (S.G.)

**Keywords:** ILC2, skin, atopic dermatitis, cancer, inflammation, Th2, IL-25, IL-33, TSLP, PD-1

## Abstract

Group 2 innate lymphoid cells (ILC2s) are lymphoid cells that are resident in mucosal tissues, especially the skin, which, once stimulated by epithelial cell-derived cytokines, release IL-5, IL-13, and IL-4, as the effectors of type 2 immune responses. This research aims to evaluate the role of ILC2s in the pathogenesis of skin diseases, with a particular focus on inflammatory cutaneous disorders, in order to also elucidate potential therapeutic perspectives. The research has been conducted in articles, excluding reviews and meta-analyses, on both animals and humans. The results showed that ILC2s play a crucial role in the pathogenesis of systemic skin manifestations, prognosis, and severity, while a potential antimelanoma role is emerging from the new research. Future perspectives could include the development of new antibodies targeting or stimulating ILC2 release. This evidence could add a new therapeutic approach to inflammatory cutaneous conditions, including allergic ones.

## 1. Introduction

The discovery of innate lymphoid cells (ILCs) dates to the early 2010s, and since then, they have been described as the innate counterpart of T cells, mirroring T-cell diversity and representing the first source of cytokines immediately available during infection, unlike the adaptive response that it takes days before it becomes effective. ILCs possess peculiar characteristics including their scarce presence in lymphoid tissues and blood circulation, being mainly localized in non-lymphoid organs and mucous surfaces [1,2]. Furthermore, ILC’s family members are characterized by classic lymphoid cell morphology but they lack the expression of cell-surface molecules that are peculiar to other immune cell types, being so far identified as cell lineage marker-negative (Lin−) cells [3,4,5,6,7]. ILCs express the common cytokine receptor γ chain (γc) and interleukin (IL)-7 receptor-α (also known as CD127), but unlike T and B lymphocytes, ILCs do not express somatically rearranged antigen receptors and, consequently, they do not exhibit any degree of antigen specificity [8]. Although they are recognized as part of innate immunity, they can elicit memory responses [8]. Host-derived signals such as food metabolites, microbic products, hormones, neuropeptides, and cytokines represent the signals sensed by ILCs through their various receptors. Although ILCs, as cells of the innate system, are involved in immune protection against pathogens, malignant cells, and noxious stimuli, recent evidence suggests that they play a role in maintaining homeostasis of the organs in which they reside [7]. Arising from a common lymphoid progenitor, ILCs have been divided into five subsets. Group 1 ILCs (ILC1s) include two subsets, natural killer (NK) cells and type 1 ILCs, and participate in type 1 immune responses by producing interferon (IFN)-γ. While NK cells are considered cytotoxic ILCs circulating in the blood or lymphatic system to counteract tumors or viral cells, in contrast, ILC1s reside in tissues such as the liver, adipose tissue, intestine, and salivary glands and they are activated by soluble cytokines including IL-15, IL-12, and IL-18. Once activated, ILC1s exert prompt, first-line responses against viral and intracellular bacterial infection through the inhibition of viral replication and promotion of macrophage activity, which in turn increases phagocytosis [9]. Moving to ILC2s, these lymphoid cells are resident in mucosal tissues including lungs, gastrointestinal tract, tonsils and, above all, the skin, and they are characterized by the expression of GATA-binding protein 3 (GATA3) [10]. Once stimulated by epithelial cell-derived cytokines, including IL-25, IL-33, and thymic stromal lymphopoietin (TSLP), they release IL-5, IL-13, and IL-4, as the effectors in type 2 immune responses; moreover, they release amphiregulin, a member of the epidermal growth factor family. Specifically, ILC2-related cytokines regulate the alternative activation of macrophages, granulocyte responses, and goblet cell hyperplasia, inducing eosinophilic inflammation and smooth muscle contractility that promote parasite expulsion [11,12,13]. ILC2-related cytokines are then involved in a plethora of pathologies including asthma, atopic dermatitis (AD), eosinophilic esophagitis, allergic rhinitis, and chronic rhinosinusitis with nasal polyps, while amphiregulin plays a role against helminthic infections and in regulating the repair of injured tissues [14]. Finally, group 3 ILCs (ILC3s) are characterized by retinoid orphan receptor γt (ROR γt) expression, thus representing the counterpart of Th17 cells. Based on the surface markers, ILC3s highly present in the intestinal mucosa, skin, lungs, and mesenteric lymph nodes, releasing IL-22, IL-17, Granulocyte-Macrophage Colony-Stimulating Factor (GM-CSF), IFN-γ, tumor necrosis factor (TNF)-α, and the heparin-binding epidermal growth factor-like growth factor (HB-EGF) [15,16], thereby generating immune responses against extracellular microbes and regulating tissue homeostasis [7]. Moreover, ILCs are highly plastic, which means that they can switch from one phenotype to another based on environmental signals, finely tuning immune responses [17]. The status and classes of the various ILCs populations are not so stiff. It has been shown that ILCs, particularly those resident in the skin, exist in a variable state and the cytokine microenvironment can induce one transition rather than another from both quiescent cells and already polarized ILCs [18]. ILC3s appear to have bidirectional plasticity depending on whether the T-box transcription factor (T-bet) or ROR γt gene expression is promoted after Notch signaling with two different effector pathways. This feature could be the result of a particular evolution of the ILC3s lineage to respond rapidly to different types of aggression [7]. Because of their variability and plasticity, ILCs can be considered an early source of cytokines in response to infection and tissue damage, promoting tissue resilience and barrier integrity of epithelial cells [19]. Finally, the activity of ILC’s subpopulations does not appear to be unrelated, as in some autoimmune diseases, a kind of balance between the levels of ILC classes has been noted. For example, in systemic lupus erythematosus, Antineutrophil Cytoplasmic Antibodies (ANCA)-related vasculitis, and rheumatoid arthritis, an increase in the ILC1/ILC2 ratio is observed in patients with active disease while, in contrast, patients with stable disease show a reduced ILC1/ILC2 ratio, suggesting an anti-inflammatory role of ILC2s that counterbalances ILC1 activity in part via IL-13 production [20].

The purpose of this review is to evaluate and shed light on the role of ILC2 in the various fields in which it is involved, mainly focusing on inflammatory cutaneous diseases, setting itself the ambitious goal of being able to hypothesize new lines of research to be able to use this lymphoid cell as a new possible therapeutic target in inflammatory diseases, whose management depends on several factors. The application of next-generation drugs that exhibit rapid onset of action with early benefits on symptomatology, using drug delivery and/or taking methods that invalidate the patient’s daily routine as little as possible, such as transdermal delivery systems, could be a breakthrough in the management of some of these chronic conditions [21,22]. 

## 2. Materials and Methods

The research was carried out using the PubMed database, inserting the keywords “ILC2” and “skin”, and mainly focusing on inflammatory skin pathologies. The preliminary research excluded previous reviews and systematic reviews, along with articles not in the English language. The results were screened and selected in the following order: title, abstract, and content.

## 3. Results and Discussion 

### 3.1. ILC2s and Cutaneous Inflammation

#### 3.1.1. ILC2s and Melanoma

As already mentioned, ILC2s are tissue-resident non-B and non-T innate immune cells that are localized in mucosal tissues, including lung, gut, and skin, mostly activated by epithelial-derived cytokines including IL-25, IL-33, and TSLP to, in turn, produce effector cytokines, including IL-5 and IL-13. Given their unique tissue localization in the mucosa, ILC2s are poised to be early players in the initiation of infiltrating tumor cells. Moreover, ILCs are highly plastic, being able to switch from one phenotype to another based on environmental conditions, consequently fine-tuning immune responses. Hence, the role of ILCs in oncology is still heterogeneous since the net effect of ILCs on the antitumor immune response will depend on the recruited subpopulation, the cytokines produced, and the signals provided by the tumor microenvironment. It is already known that ILC2s through their plasticity, under certain conditions, favor the onset and proliferation of tumors, including breast, gastric, and prostate cancer, through the production of IL-4 and IL-13 and the immunosuppressive functions of Tregs, produced by amphiregulin [23,24,25]. Amphiregulin, in turn, promotes the growth of the tumor-expressing epidermal growth factor receptor (EGFR) [26] as well as tissue invasion and metastases processes [27]. ILC2s also interacted with myeloid-derived suppressor cells (MDSCs), which exert immunosuppressive activity. ILC2s induced the accumulation of monocytic MDSCs in acute promyelocytic leukemia and bladder cancer through the production of IL-13 [28]. ILC2s induced by IL-33 induced tumor growth in both a mouse model of triple-negative breast cancer and pancreatic ductal adenocarcinoma. Finally, ILC2s can also act directly on anticancer cells, including NKs, to repress antimetastatic functions. On this topic, Long et al, in a Rag1−/− mouse model, characterized by the lack of T and B lymphocytes, demonstrated that systemic administration of recombinant IL-33 in melanoma cells led to an upregulation of ILC2 levels, through its receptor ST2, which in turn inhibited NK cell activation and cytotoxicity, thus favoring tumor growth. [29]. Similarly, Schuijs et al., in a lung metastasis model, demonstrated that ILC2s were activated in an IL-33-dependent way, which caused NK inhibition and subsequent INF-γ production and cytotoxic function suppression, leading to cancer spreading [30]. However, as evidence of the dual role of ILC2s, other studies reported that the IL-33/ILC2 axis can exert tumor suppressive activity in hepatocellular carcinoma, pancreatic tumors, and melanoma [31,32]. Among the tumoral conditions, melanoma is a highly aggressive cancer that spreads from primary cutaneous or mucosal sites to internal organs throughout the human body [33]. Since the prognosis of melanoma becomes very unfavorable in case of metastasis, new therapies have been developed to improve patients’ outcomes. Among molecularly targeted approaches, Nivolumab is an antibody that inhibits programmed death-1 receptor (PD-1), enhancing median overall survival. PD-1 binds to ligands PD-L1 or PD-L2, acting as an inhibitory immune checkpoint, to negatively regulate immune cell activation and effector function [34]. Howard et al. demonstrated that ILC2s can express PD-1 when activated by IL-33. PD-1 dramatically reduced ILC2 proliferation, viability, and effector function, while inhibition of PD-1 conversely resulted in increased total ILC2 number, cytokine production, and overall cellular function [35]. In fact, high ILC2 infiltration in human melanoma was associated with a good clinical prognosis. ILC2s stimulated the production of GM-CSF, which, in turn, coordinated eosinophil recruitment and activation to enhance antitumor responses. Tumor-infiltrating ILC2s expressed PD-1 that downregulated their intratumoral accumulation, proliferation, and antitumoral functions. In vivo, it has been proposed to activate IL-33-driven ILC2s and block PD-1 with the aim of blocking the ILC2-related inhibition mechanism of antitumor activity. From this, we deduce a potential role of ILC2s in a hypothetical synergistic approach with the immunotherapies already approved [36]. Using a melanoma model, Wagner et al. demonstrated that tumor cells exerted immunosuppressive activity against ILC2s via the accumulation of lactic acid in the tumor microenvironment. Acid lactic inhibited ILC2 proliferation and cytokine production, also reducing in vitro survival. The authors demonstrated that in knockdown lactate dehydrogenase A B16F10 melanomas (LDHAlow), the number of intratumoral ILC2s increased proportionally. Furthermore, demonstrating the stimulating action of IL-33, this interleukin led to ILC2 expansion in LDHAlow B16F10 tumors, in association with the presence of eosinophils, which consequently slowed tumor growth. Finally, higher expressions of IL-33 and an eosinophilic marker, SIGLEC8, were associated with improved overall survival in patients with cutaneous melanoma, suggesting the role of the IL-33/ILC2/eosinophil axis in antimelanoma immunity. The data presented suggest that specifically targeting the lactic acid produced by the tumor would consequently increase the ILC2 intratumoral infiltrate, thus interrupting the immunosuppressive mechanism. [37]. As a further demonstration of the antitumor role of the IL-33/ILC2 axis, Okuyama et al., in a mouse model of melanoma, demonstrated that ILC2s, once activated by IL-33 via their ST2 receptor, induced CD8+ T-cell expression and infiltration into the tumor. Indeed, in mice treated with IL-33, ILC2s expressed higher levels of the ligand (OX40L) while CD8+ T cells expressed the OX40 receptor. This binding induced the action and proliferation of CD8+ T cells against melanoma cells. Moreover, the in vivo blockade of the OX40L-OX40 interaction, by the administration of anti-OX40L antibodies, inhibited IL-33 antitumor effect. Thus, the IL-33/ILC2 axis promotes CD8+ T-cell responses through the OX40/OX40L binding and exerts an antitumor effect, representing a possible new research line for the study of new drugs that selectively favors the OX40-OX40L binding [38].

**Figure 1:**
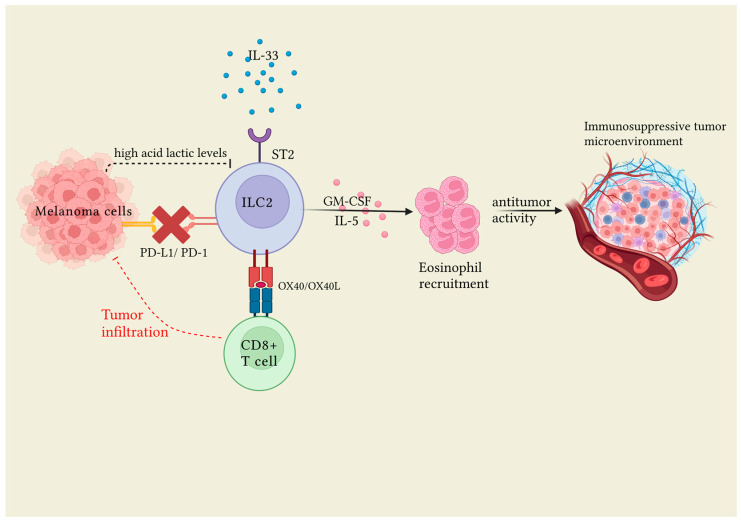
Following cell injury, IL-33 is released and induces ILC2 proliferation via the ST2 receptor expressed on the cell surface. ILC2s in turn, through GM-CSF and IL-5, induce the activation of eosinophils, which play an antitumor role. In order for ILC2s to be activated it is necessary that the PD1 link, expressed on ILC2s, with PD-L1 expressed on melanoma cells is inhibited. IL-33 induced the expression of OX40L and OX40 in ILC2s and CD8+ cells, respectively, thus enhancing antitumor effect through the infiltration of CD8+ cells into the tumor. Furthermore, high levels of lactic acid released from the tumor play a role in inhibiting ILC2 activity. Created with BioRender.com.

As already discussed for melanomas, IL-33 was found to activate tumor ILC2 (TILC2) and CD8+ T cells in orthotopic pancreatic skin tumors to limit pancreatic-specific tumor growth. Resting and activated TILC2s expressed the inhibitory checkpoint receptor PD-1, and TILC2s further expanded by blocking PD-1 to improve tumor control. PD-1 blockade, expressed on both resting and activated TILC2s, acted directly on TILC2s by expanding them to enhance antitumor immunity and the efficacy of anti-PD-1 immunotherapy. This allowed us to identify activated TILC2s as new major targets of immunotherapy and, therefore, as possible novel anticancer immune cells for orthotopic pancreatic skin tumor therapy [32]. Finally, in hepatocarcinoma, the specific changes in cytokines modified the ILC2 composition, thus shifting their actions toward an antitumoral function [31]. Specifically, decreasing levels of IL-1B, IL-23A, IL-17A, IL-2, IL-15, IL-12A, IL-9, IL-33, and IL-4 corresponded to a better prognosis [31]. Figure 1 represents the central role of ILC2s in malignant melanoma.

#### 3.1.2. ILC2s and Sistemic Sclerosis

Recently, the presence of activated ILC2s has been detected in inflammatory diseases, not type 2, on an autoimmune basis, such as systemic sclerosis, in particular, in the diffuse form and in forms with lung involvement (pulmonary fibrosis) [39]. In scleroderma, skin remodeling is mainly supported by the proliferation of fibroblasts with collagen deposition, resulting in skin thinning and in the loss of skin elasticity, which is typical of patients affected by this pathology [40]. The trigger of profibrotic mechanisms of the cutaneous fibroblast could be partly given by the interaction between ILC2s and transforming growth factor (TGF)-β. TGF-β is a mediator over-expressed in scleroderma and systemic sclerosis (SSC) patients and downregulates the expression of Killer Cell Lectin-Like Receptor G (KLRG1). KLRG1+ ILC2s circulating could migrate to the skin and pass to their profibrotic form, KLRG1− ILC2s, causing an imbalance between profibrotic and antifibrotic mediators and activating profibrotic mechanisms in the skin fibroblast. In addition, KLRG1− ILC2s have an impaired ability to secrete IL-10, which contributes to the profibrotic effect. Finally, ILC2s that are stimulated by TGF-β have a higher expression of leukotriene synthetase and can potentially produce leukotriene C4, which is a strong stimulus of collagen synthesis by dermal fibroblasts [41]. The pathway involved would appear to be mast cell-Th9-ILC2s that are already detected in cystic fibrosis. In patients with systemic sclerosis, elevated levels of IL-9 were found in accordance with elevated circulating levels of Th9 lymphocytes. Following the addition of IL-9 in vitro experiments, mast cells have undergone proliferation and activation with IL-2 production, which is known to activate dermal ILC2s [42].

#### 3.1.3. ILC2s and Parasites Infections

Recently, a growing interest has been placed on the study of the metabolic requirements of classical T cells at various stages of activation. Moving on to helminthic infections, it was shown that ILC2s, which represent an important mediator of barrier immunity, had a dependent fatty acid metabolism for IL-13 production, especially in conditions of vitamin A deficiency. From this, it can be deduced that the selective use of fatty acids in the context of deficiency and helminth infection represents an immune adaptation mechanism to maintain the integrity of the skin barrier [43]. Switching to another inflammatory cutaneous disease, rosacea is characterized by the overgrowth of Demodex folliculurom. Demodex mites, commensal parasites of the hair follicles, required ILC2s and IL-13 to colonize the hair follicle in a mouse model. ILC2s upregulated IL-13, which in turn, attenuated epithelial proliferation at anagen initiation, whereas in the absence of ILC2s, colonization of Demodex led to the increased epithelial proliferation and replacement of gene programs for repair by aberrant inflammation, leading to loss of barrier function and follicle depletion. The loss of canonical alarmins attenuated ILC2 response, suggesting a prominent role for alarmins in regulating basal ILC2’s numbers and tone, but revealing alternative pathways by which ILC2s react to tissue perturbations induced by this commensal. This suggests a key role for ILC2s and IL-13 in the skin, an immune checkpoint that supports skin integrity and limits pathological infestation [44].

#### 3.1.4. ILC2s and Hidradenitis Suppurativa

Among the inflammatory cutaneous diseases, a growing interest especially from pharmaceutical companies has been poned in hidradenitis suppurativa (HS), which is a condition involving the apocrine gland leading to the development of abscesses, sinus tracts, and scars [45]. Petrasca et al. analyzed, by multiparameter flow cytometry, the frequencies of ILC subsets in skin and peripheral blood mononuclear cells of HS patients compared with healthy controls and psoriasis patients. In HS patients, both lesional and non-lesional skin significantly increased in the absolute ILC numbers compared to control skin. The authors concluded by hypothesizing that ILCs could participate in the pathogenesis of HS, which would open a further line of research for the development of new drugs targeting the pathways involved in the pathogenesis. To date, in fact, HS represents a challenge for the clinician, as adalimumab is the only biological drug approved so far [46].

#### 3.1.5. ILC2s: Skin Wound Healing and Skin Immune Tolerance 

Another chapter in which interest in the immune response is increasingly developing concerns skin wounds. While ILC2s have been demonstrated to play a pathological role in the context of chronic dermal inflammation, it remains possible that these cells may play a host-protective tissue reparative role in restoring cutaneous barrier functions following acute injury. In a mouse model and human skin examples, it was demonstrated that skin injury promotes an IL-33-dependent ILC2 response and that abrogation of this response impairs re-epithelialization and efficient wound healing. Together, these results indicate that IL-33-sensitive ILC2s are an important link between the skin epithelium and the immune system, acting to promote the restoration of skin integrity after injury [47]. The mechanisms underlying inflammation and skin remodeling could be traced back to the balance between pro-inflammatory and anti-inflammatory and pro-fibrotic and antifibrotic factors, and this could suggest the involvement of other cells and cytokines of the innate immune response, as it has been seen in autoimmune diseases through the study of the relationship between various ILC populations. The role of IL-33-driven ILC2 response has also been proven in promoting skin wound healing in diabetic mice treated with exogenous IL-33. Initially, skin damage stimulated the innate proinflammatory response and inflammasome activation, while in the second stage, mechanisms that limited the inflammatory response and promoted the transition to a reparative response occurred. IL-33 could coordinate the reparative mechanism by activating ILC2s and triggering anti-inflammatory responses [48]. In fact, the activation of the inflammasome machinery and caspase-1 also led to the cleavage and maturation of cytokines that counteract the pro-inflammatory effects, such as IL-37. The role of this recent cytokine is still being defined, and the existence of an IL-33/IL-37 regulatory axis at the skin level has been proposed [49]. In synergy with the role of resident ILCs, this potential axis could be involved in the genesis of skin inflammation and consequently provide a possible therapeutic window. 

Evidence suggested that ILC2s are involved in tissue repair and that they facilitate immune tolerance after skin transplantation. In a human skin xenograft model, the effect of AJI-100 as prophylaxis of rejection after tissue transplantation was evaluated. It has been hypothesized that the combination of Janus kinase (JAK)-2 and Aurora kinase A can completely control alloreactive human T cells, an effect that the two molecules are unable to achieve alone, thus improving allograft survival. The dual blockade significantly reduced rejection due to ILC2s, which are involved in tolerance, tissue regeneration, and repair. AJI-100 maintains adequate levels of phosphorylated Signal transducer and activator of transcription 5 (pSTAT5), which in turn regulates the expression of GATA3, which is required for ILC2 development. These pieces of evidence confirm that ILC2s play a protective role against GVHD [50]. In fact, allogeneic hematopoietic stem cell transplantation (HSCT) is often complicated by graft versus host disease (GVHD), which leads to severe epithelial impairment. In a study conducted on 51 patients affected by leukemia and evaluating the effects of induction chemotherapy, conditioning radiochemotherapy, and allogeneic HSCT recovery of circulating ILCs, it was highlighted that the proportion of ILCs expressing markers of activation, proliferation, and skin tissue homing was associated with reduced susceptibility to therapy-related mucositis and acute GVHD, which could hint that ILC recovery and treatment-related tissue damage are related to and influence the development of GVHD [51]. A mouse model conducted by Bruce et al. highlighted that third-party ILC2 cells significantly improved mice survival after allo-HSCT when administered at the time of transplantation. ILC2 cells when used therapeutically were effective but less robust compared with the administration of ILC2 cells to prevent GVHD [52].

#### 3.1.6. ILC2 and Mastocytosis

Finally, the role of ILC2s has been evaluated in mastocytosis, a hematological disease characterized by aberrant mast cells due to gain-of-function mutations in the KIT receptor, which can also have cutaneous manifestations. On this topic, 21 patients with systemic mastocytosis were compared to 18 healthy controls and it was found that patients with skin involvement and pruritic symptoms had higher ILC2 levels, independent of serum tryptase levels. The authors suggested that ILC2 activation could lead to a cytokine milieu, such as atopic dermatitis, which promotes inflammation and skin symptoms [53]. Table 1 summarizes the main findings about the involvement of ILC2s in inflammatory cutaneous diseases.

### 3.2. ILC2s and Allergic Cutaneous Inflammation 

ILC2s are the most represented population of ILCs in the skin, playing a pivotal role in cutaneous inflammation. Dermal ILC2s (dILC2s) have been demonstrated to have at least two functional states: steady-state and stimulated dILC2s. Steady-state ILC2s play an immuno-regulatory action, producing IL-13 and thus regulating mast cell activity, while stimulated dILC2s, which assume a pro-inflammatory activity, recruit eosinophils and activate mast cells [54]. ILC2 activation depends on stimuli, identified as pathogen-associated molecular pattern molecules (PAMPs) or alarmins, which bind to specific receptors expressed on the surface of those cells and initiate responses framed within innate immunity. As previously discussed, the alarmins that are responsible for ILC2 activation are the so-called epithelium-derived cytokines: IL-33, TSLP, and IL-25. While TSLP seems to play a major role in the activation of cutaneous ILC2s, IL-33 is responsible for the migration of MHCII+-ILC2s to lymph nodes, where ILC2s take part in adaptive immune response [55]. In addition to the above-mentioned cytokines, ILC2s are also activated and regulated in both positive and negative directions by other cell types and by circulating cell-derived cytokines. A crosstalk between keratinocytes and ILC2s has been highlighted in chronic allergic skin inflammation, identifying the leading roles of IL25 and IL-18. IL-25 produced by keratinocytes stimulates cutaneous ILC2s, consequently activating T helper cells, increasing the production of IL-13 and T-cell attracting chemokines and promoting epidermal hyperplasia [56]. Regarding the role of IL-18, in mouse models, IL-18 deficiency resulted in poor activation of IL-18R-expressing cutaneous ILC2s. Subpopulations of ILC2-expressing receptors for IL-18 could be partly responsible for the most severe forms of allergic skin inflammation and increased levels of IL-18 that correlate with the severity of dermatitis. Finally, IL-18 and IL-33 action on ILC2s would appear to be closely related, since the respective receptors are simultaneously expressed on the surface of certain ILC2 subpopulations and, furthermore, the genes encoding these two receptors are both located in a range of the genome that has been associated with an increased risk of developing allergic diseases. These observations can have interesting therapeutic implications, looking at IL-18 as a new target in regulating type 2 skin inflammation [57]. Moreover, recent studies have identified a close interaction between basophils and ILC2s. Basophils promote the activation and immunological mechanisms of ILC2s promoting IL-4 production through a direct mechanism. In addition, IL-4 could probably play a chemotactic action toward ILC2s by increasing their responsiveness to epithelium-derived cytokines, which in turn, regulate cytokine production by basophils [58,59]. Other mediators released by basophils could also contribute to these mechanisms, such as leukotrienes [58]. Additionally, mast cells influence ILC2s in allergic cutaneous inflammation, through cytokines’ releasing and through ligand–receptor interaction [54]. Prostaglandin 2 (PDG2) released by mast cells binds to its receptor CRTH2 expressed on ILC2 surface, while ILC2-produced IL-13 could reduce mast cell activation [59]. 

ILC2 activity in skin inflammation is also downregulated by Treg expressing the nuclear receptor RORα, blocking the expression of the gene encoding for IL-4 and the conversion of Treg to IL-4-producing effector cells. RORα was demonstrated to be more expressed in circulating Tregs than in cutaneous and intestinal ones: the former regulates local inflammatory processes while the latter has a remote regulatory action in the skin, given the known correlation between the cutaneous barrier and intestinal barrier. This evidence supports the hypothesis that certain polymorphisms in the gene encoding for RORα may underlie the genesis of Th2/ILC2-mediated skin inflammatory diseases [60]. Among the inflammatory diseases, allergic contact dermatitis (ACD) is an allergic skin condition that develops following contact with a haptene to which the subject is sensitized, mounting a type IV immune response (delayed and T-cell mediated). According to a recent study, ILC2 activation would be critical for type 2 skin inflammation in ACD, and its absence would promote a type 1 immune response [61]. There is a crosstalk between ILC2s and CD4+ T lymphocytes through the action of MHCII and IL-2: only stimulation by IL-2 seems to be sufficient for ILC2 activation [54]. Therefore, depletion of ILC2s would increase the availability of IL-2 through other pathogenetic mechanisms underlying contact hypersensitivity reactions. In addition, ILC2s might have a counter-regulatory action in contact hypersensitivity reactions mediated by T and NK lymphocytes [61]. Moreover, ILC2-producing IL-10 with regulatory action in allergic lung inflammation was identified. However, this function has not yet been identified for cutaneous ILC2s, and further studies are needed [61]. Finally, skin allergic diseases with an underlying mixed IgE-mediated and T-cell-mediated immunologic mechanism have recently been identified, and ILC2s could be the potential mediator of these immunologic reactions. A study conducted by Shane et al. suggested didecyldimethylammonium chloride (DDAC) to be responsible for a cutaneous inflammatory reaction mixed-type, thus showing an early increase in IL-33 and TSLP in the 12 hours following exposure to DDAC [62]. Moreover, a downregulation of E-cadherin, which inhibits Th2 cytokine production by ILCs, has been observed, assuming that this reduction may be responsible for ILC2 activation and Th2 cytokine release.

The inflammatory role of ILC2s has also been proven in Drug Rash with Eosinophilia and Systemic Symptoms (DRESS) syndrome: the stimulation of ILC2s by IL-33 is responsible for skin manifestations, eosinophilic inflammation, and poor response to corticosteroids. However, these mechanisms not only contribute to inflammation but are also involved in reparative mechanisms through the expression of ST2 and ILCS2 rapid response to IL-33 stimulation. In this syndrome, ILC2s releasing epidermal growth factors and activating eosinophils could play a role in wound healing, skin remodeling, and fibrosis [63]. Monoclonal anti-TSLP antibodies reduce ILC2 activation and resolve the resistance that ILC2s develop toward steroids as a result of TSLP action, making this drug a viable therapeutic option for inflammatory diseases that are resistant to corticosteroid therapies [64]. Table 2 summarizes the main findings about the role of ILC2s in allergic cutaneous inflammation.

#### Focus on Atopic Dermatitis

Excessive type 2 immune responses represent the hallmark of AD in humans and mouse models [65]. Studies of induced type 2 inflammation in mice and naturally occurring human AD showed that Th2 cells and ILC2s express the master transcription factor GATA3 and produce type 2 cytokines, including IL-4, IL-5, and IL-13. These mediators elicit pruritus and skin inflammation, IgG1 and IgE production, and decreased skin barrier integrity, which drives AD pathogenesis. A study that identified a canine immune cell type, with similar phenotypes and functions to human and murine ILC2s, and canine Th2 cells ex vivo, using an anti-GATA3 antibody, observed that in atopic dogs, the number of Th2 cells was high, while that of ILC2s was not. Elevated frequencies and numbers of Th2 cells but not ILC2s in peripheral blood were associated with chronic canine atopy [66]. A mouse model using transgenic binary mice that are constitutively deficient in conventional dendritic cells (cDCs) demonstrated that congenital cDC deficiency not only exacerbated AD-related inflammation but also resulted in immune alterations such as increased levels of granulocytes, ILC2s, and B cells. cDCs deficiency likely resulted in disruption of a homeostatic feedback loop mediated by Fms-related tyrosine kinase 3 ligand (Flt3L). In addition, increased skin colonization of Staphylococcus aureus has been observed. Thus, cDCs have a role in maintaining immune homeostasis, maintaining skin barrier integrity, and thereby avoiding disease exacerbations [67].

Additionally, in humans, AD has been linked to ILC2s, which have been found to be markedly increased in the skin of affected patients [68]. ILC2 levels were markedly upregulated in the lesional skin compared to skin samples collected from healthy controls [69,70,71]. Specifically, studies identified ILC2s for the first time in both mice and humans [54,69] and found that they were present in high concentrations in lesional skin [69]. Moreover, in the mouse model, ILC2s, which are mostly dependent on TSLP, were fundamental for the development of AD-like disease, through the production of IL-5 and IL-13 [54,69]. The presence of ILC2s and ILC3s in healthy skin, producing IL-13 and IL-22, respectively, and almost no ILC1s, has been detected in vivo. Characterization of ILC2s found that they could be expanded in vitro with high doses of IL-2 without loss of phenotype or function and maintain their ability to produce IL-13 after in vitro culture. IL-25, and to a lesser extent, IL-33, enhanced TSLP’s ability to stimulate ILC2s, a result that may have implications considering the use of these pathways as targets in AD treatment [72]. In addition to their role in innate immune response, ILC2s could also activate Th lymphocytes through the expression of MHC-II and peptide antigens. In a human model, it was found that in the presence of AD, TSLP-induced ILC2s expressed Cluster of Differentiation 1a (CD1a), which, in turn, activated Th lymphocytes. In addition, ILC2s presented endogenous lipid antigens to CD1a-reactive T cells. This pathway was also used by lymphocytes to detect Staphylococcus aureus and promote skin inflammation [73]. Moreover, ILC2s lack rearranged antigen-specific receptors, although they can be influenced by many soluble factors, such as cytokines and lipid mediators. On this topic, an in vitro model attempted to evaluate the expression and functional properties of the natural cytotoxicity receptor NKp30 on ILC2s. Cultured ILC2s subset expressing NKp30, after interaction with its activator ligand B7-H6, rapidly produced type 2 cytokines. Furthermore, increased expression of B7-H6 has been observed in lesional skin biopsies of AD patients. Finally, keratinocytes’ incubation with pro-inflammatory and type 2 cytokines increased B7-H6 expression leading to the overproduction of ILC2s. The NKp30-B7-H6 interaction can be considered a novel cell contact mechanism that mediates ILC2 activation and identifies a potential target for the development of novel therapeutic choices for AD [74].

IL-33 is a proinflammatory cytokine of the IL-1 family, constitutively expressed in the nucleus of epithelial or endothelial cells, that participates in pathological processes of allergic nature, including asthma, conjunctivitis, and AD [49]. In cellular damage or stress, IL-33 is rapidly released as an endogenous danger signal or alarm to alert and activate the innate immune system [75]. During its effector action, IL-33 binds to its receptor, ST2, expressed in Th2 cells, basophils, mast cells, and ILC2s [76]. A mouse model evaluated the role of ILC2s correlated to IL-33 in the pathogenesis of atopic lesions. The depletion of ILC2 cells in IL33tg mice almost completely suppressed the development of inflammation, demonstrating the central role of ILC2s in immune processes [77]. Thus, IL-33-induced dermatitis is dependent on ILC2s, which are increased in human AD skin lesions, which can open a line of research to specifically target upstream ILC2s. To confirm the determinant role of IL-33, Imai et al. generated transgenic mice expressing the mouse IL-33 gene. In 6–8 weeks, the mice developed pruritic dermatitis with histological features characterized by thickening of the epidermis and intense eosinophilic infiltrate and mast cells in injured skin. The percentage of ILC2 cells that produced IL-5 was significantly increased in lesional skin, peripheral blood, and regional lymph nodes. These results suggest that IL-33 expression in the skin activates an immune response involving ILC2s and that this process could play a pivotal role in AD-related skin inflammation pathogenesis [78]. As already specified, IL-33 induces the expression of type 2 cytokines and amphiregulin, increasing ILC2 migration. On this topic, Salimi et al. demonstrated that the binding of E-cadherin, a fundamental adhesion protein, to human ILC2s dramatically inhibited the production of IL-5, IL-13, and amphiregulin. Interestingly, downregulation of E-cadherin is characteristic of filaggrin insufficiency, a cardinal feature of AD. Thus, in the absence of E-cadherin, ILC2s led to increased production of type 2 cytokines [70]. This was also seen in a BALB/c mouse model, where ILC2s in the skin promoted inflammation, which was in turn induced by IL-25, IL-33, and, to a lesser extent, by TSLP. Indeed, the greatest reduction in skin inflammation was observed in mice lacking IL-25. Moreover, filaggrin (Flgft/ft) mutant mice developed spontaneous skin inflammation accompanied by increased levels of ILC2s, production of IL-1β, and other inflammatory cytokines, which is similar to what occurs in human AD. Wildtype and Flgft/ft mice differ significantly in the composition of their microbiomes. Contrary to expectations, ILC2 deficiency did not improve AD in Flgft/ft mice, which was also independent of IL-4, IL-5, and IL-13, but required the signaling of IL-1β and IL-1R1, which therefore also play a fundamental role in the development of inflammation. [79]. Several pieces of evidence now assert that AD is supported by an imbalance between antioxidant factors and oxidants, which causes oxidative stress [80]. In the push for increasingly popular phytopharmaceuticals, an attempt has been made to find natural products protecting against stress induced by toxic substances [81]. In this regard, Celastrol, belonging to a quinone methyl compound, isolated from Trypterygium Wilfordii Hook, exerts anti-inflammatory and antiallergic effects by inhibiting the activity of mast cells and basophils. Topical application of celastrol significantly improved atopic dermatitis symptoms induced by house dust mite (HDM) in NC/Nga mice, as determined by dermatitis score and histological assessment. Celastrol decreased the levels of TSLP, ILC2s, and Th2-cytokines in AD skin lesions of HDM-stimulated NC/Nga mice, thus suggesting that the reduction in TSLP levels and the number of ILC2 cells could ameliorate AD-related symptoms [82]. Additionally, xanthophyll, which is mainly contained in marine plants, has been demonstrated to play an antioxidant role against AD on a NC/Nga mice model. The authors compared the anti-itch effect of the extract with tacrolimus, observing that the former significantly reduced symptoms compared to the latter, through the presence of ILCreg suppressing the immune response. [83]. The possible role of infection in the elicitation or inhibition of AD has been suggested in a study investigating whether chronic infection with Toxoplasma gondii could modulate the development of AD using chronically infected mice sensitized by repeated epicutaneous ovalbumin administration. Chronic infection with Toxoplasma gondii appeared to prevent AD development through a reduced susceptibility that appeared to result from changes in type 2 innate immune response, with reductions in IL-4, IL-5, and ILC2s [84]. In an AD mouse model, immune tolerance was induced by oral administration of ovalbumin with subsequent epicutaneous sensitization by repeated application of ovalbumin. The induction of oral tolerance resulted in a reduction in the inflammatory response, through the inhibition of ILC2 levels [85]. In conclusion, all the evidence reported so far agrees in considering ILC2s of central importance in the pathogenesis, severity and prognosis of atopic disease. Its close relationship with IL-33 may open a new line of research for the study of antibodies that selectively inhibit ILC2/IL-33 binding, through the ST2 receptor, thus blocking the type 2 inflammatory cascade upstream that characterizes atopic dermatitis.

**Figure 2:**
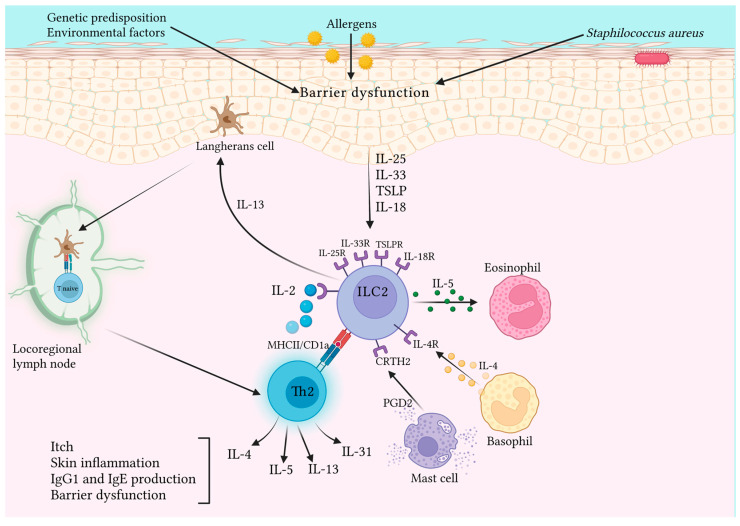
Following skin barrier alteration, damaged keratinocytes release epithelial-derived cytokines, including IL-25, IL-33, IL-18, and TSLP. ILC2s expressing the receptors for these cytokines (IL-25R, IL-33R, IL-18R, and STLP-R) on their surface are then activated, in turn, releasing Th2 cytokines. Among these, IL-5 determines the activation of eosinophils, while IL-13 stimulates epidermal Langerhans cells (LC), which in turn, migrate to regional lymph nodes to prime naïve T cells by antigen presentation via MHCII to promote the development of Th2 cells. Th2 cells produce IL-4, IL-5, IL-13, and IL-31. All these cytokines promote itch, skin inflammation, IgG1 and IgE production, and maintenance of barrier dysfunction, thus rendering this condition chronic. Finally, ILC2s can act as antigen-presenting cells for Th2 effector cells through antigen presentation via MHCII and/or CD1a prompting them to produce IL-2, which in turn, sustains ILC2 activation and survival. Created with BioRender.com.

Table 3 summarizes the main findings about the role of ILC2 in AD. Figure 2 represents the main pathways and cytokines involved in AD pathogenesis.

### 3.3. Possible Therapeutic Uses of ILC2s in Inflammatory Pathologies

The studies reported so far suggest a future potential therapeutic role of ILC2s in two main pathologies, cutaneous melanoma and atopic dermatitis, which express two opposite poles of inflammation. In melanoma, albeit in single studies conducted on mice, the role of the IL-33/ILC2/eosinophil axis has been highlighted [35,36,37]. It was also observed that ILC2s expressing PD-1, which are stimulated by IL-33, bind to cancer PD-L1, which in turn inhibits the anticancer effect linked to ILC2s. Hence, the blockade of PD-1 has been proposed, together with the therapies that are already approved, with the contextual stimulation of ILC2s via IL-33 [38]. An indirect antitumor action has also been proposed through the stimulation of the binding between ILC2s and T cells by OX40 and OX40L, which increased the T cells’ intratumoral infiltration. In fact, selective blockade of OX40L by a specific antibody inhibited the antitumor action of the IL-33/ILC2s axis [38]. Moving to atopic dermatitis, the potential therapeutic role of ILC2s derives from their central position in the pathogenesis of the disease. In fact, the monoclonal antibodies approved so far (including anti-IL-4/IL-13, and anti-IL-13) and those in the process of being approved (such as anti-IL-31) target molecules further downstream of ILC2s [80]. Furthermore, the studies reported so far have highlighted the pivotal role of IL-33 in the stimulation of ILC2-mediated inflammatory action. Scientific research is now focusing more and more on inflammation mediators. In this regard, a monoclonal antibody (ASP7266) against the TSLP receptor (TSLPR) expressed on the ILC2 surface has been proposed for the treatment of allergic pathologies. In vitro, ASP7266 confirmed its inhibitory effects on TSLP-induced cell proliferation and cytokine production for both CD4 T cells and ILC2s [64]. Moreover, knowing that TSLP confers steroid resistance to ILC2s [86], ASP7266 may be a valid therapeutic choice for steroid-resistant inflammatory diseases, including severe AD and asthma. Similar to what happens in AD, in asthma, scientists tried to target upstream inflammation mediators, including TSLP. Clinical trials have been conducted for the anti-TSLP antibody, tezepelumab (CSJ-117 and MK-8226), and for the anti-TSLPR antibody (RG7258). Tezepelumab has been approved for patients with severe asthma as an add-on maintenance treatment in adults and adolescents of 12 years of age and older who are inadequately controlled, despite high doses of inhaled corticosteroids plus another medicinal product for maintenance treatment [87]. Most of the biological therapies approved for severe asthma are adapted to T2 endotype asthma, particularly eosinophilic asthma. Patients with severe asthma of non-T2 endotype have limited therapeutic options in the field of biological therapy. In this regard, the study ZENYATTA, phase 2b randomized, double-blind, dose-ranging, and placebo-controlled, has recently shown that astegolimab, a human IgG2 monoclonal antibody that selectively blocks the IL-33 receptor, ST2, was able to significantly decrease asthma exacerbations in patients with severe asthma, being expressed on the cell surfaces that are not exclusive to T2-high inflammation, including ILC2s [88]. Astegolimab could act upstream of the inflammatory process for both T2 and non-T2 asthma as effector cells in both asthma’s endotypes express the ST2 receptor for IL-33 [89]. Targeting a receptor expressed by Th2 inflammation cells that further amplifies the Th2 response appears to be a more valid and effective therapeutic strategy since the various cell species of innate and adaptive immunity responsible for Th2-mediated inflammation are blocked. In this way, the inflammatory process responsible for the pathogenesis of asthma is blocked upstream rather than the action carried out by specific monoclonal antibodies targeting a single mediator. This strategy could also be applied to other diseases whose pathogenesis depends on cytotypes and mediators of the Th2 profile. Finally, the possibility of applying this therapeutic strategy in other inflammatory diseases in whose pathogenesis ILC2s plays a central role cannot be ruled out; however, no clinical trials have been identified so far in this regard. Table 4 highlights the main findings about the potential therapeutic role of ILC2s in melanoma, AD and cutaneous type 2 inflammation.

## 4. Conclusions and Future Perspectives

ILC2s are lymphoid cells that are resident in mucosal tissues, especially the skin, which, once stimulated by epithelial-derived cytokines, release IL-5, IL-13, and IL-4, as the effectors of type 2 immune responses. Our research showed that ILC2s play a central role in the pathogenesis, prognosis, and severity of several inflammatory cutaneous diseases, especially atopic dermatitis. However, the newest aspect, as well as the least investigated in the literature, is the role played by this subset of innate cells in melanoma. In fact, in several tumors, ILC2s play a dual role, both facilitating tumor progression and inhibiting tumor growth, which is further evidence of the plasticity of these cells. Nevertheless, in cutaneous melanoma, recent studies have shown that ILC2s, once stimulated by IL-33 and after binding with its ST2 receptor, play an antitumor role through the release of GM-CSF and IL-5, which in turn cause the recruitment of basophils, thus inducing an unfavorable tumor environment. This evidence can open doors to future therapeutic perspectives. In fact, while atopic dermatitis research should be aimed at the inhibition of ILC2s to block the release of the Th2-type cytokines that induce the disease, ILC2 release should be stimulated in melanoma instead, through IL-33 to favor their antitumor action. Furthermore, it would also be useful to simultaneously block PD1 expression by ILC2s so as not to be downregulated by tumor cells through binding to PD-L1. These results could be useful in developing new therapeutic approaches that could include the use of target therapy, possibly together with already validated drugs, to achieve amelioration of the disease and improve the quality of life of affected patients. However, further research is needed to explore and elucidate this potential research field, which can represent a sliding door in the treatment of chronic pathologies, such as atopic dermatitis, and pathologies with a poor prognosis, such as melanoma.

## Figures and Tables

**Table 1:** The role of ILC2s in inflammatory cutaneous diseases: systemic sclerosis, hidradenitis suppurativa, parasite infections, wound healing, graft versus host diseases, and mastocytosis.

Authors	Year	Topic	Study Characteristics
Laurent et al. [41]	2021	Sistemic sclerosis	Killer Cell Lectin-Like Receptor G (KLRG1)-Group 2 Innate lympohid cells (ILC2s) have the following: - Profibrotic action; - An impaired ability to secrete interleukin (IL)-10, which contributes to the profibrotic effect; - Higher expression of leukotriene synthetase, which is a strong stimulus of collagen synthesis by dermal fibroblasts.
Guggino et al. [42]	2017	Sistemic sclerosis	Following the addition of IL-9 in in vitro experiments, mast cells have undergone proliferation and activation with IL-2 production, which is known to activate dermal ILC2s.
Wohlfahrt et al. [39]	2016	Sistemic sclerosis	Detection of ILC2s in diffuse form and in forms with lung involvement (pulmonary fibrosis).
Petrasca et al. [46]	2023	Hidradenitis suppurativa	Hidradenitis suppurativa lesional and non-lesional skin significantly increased in the absolute innatel lymphoid cell (ILC) numbers compared to control skin, suggesting the role of ILCs in hidradenitis pathogenesis.
Ricardo-Gonzalez et al. [44]	2022	Demodex folliculorum	ILC2s elaborated IL-13 that attenuated air follicles and epithelial proliferation; in their absence, Demodex colonization led to increased epithelial proliferation and replacement of gene programs for repair by aberrant inflammation, leading to the loss of barrier function and follicle depletion.
Wilhelm et al. [43]	2016	Helminthic infections	ILC2s have a dependent fatty acid metabolism for IL-13 production, especially in conditions of vitamin A deficiency.
Rak et al. [47]	2016	Wound healing	A mouse model and human skin samples model demonstrated that skin injury promotes an IL-33-dependent ILC2 response while the abrogation of this response impairs re-epithelialization and efficient wound closure.
Li et al. [48]	2022	Wound healing in diabetic mice	IL-33-driven ILC2 response promotes wound repair by activating pro-inflammatory processes during the first phase and subsequently limiting inflammation by promoting the transition to reparative mechanisms.
Walton et al. [50]	2021	Skin graft rejection	The dual blockade of janus kinase (JAK)-2 and Aurora kinase A reduced rejection due to ILC2s, which are involved in tolerance, tissue regeneration, and repair. AJI-100 maintains adequate levels of phosphorylated Signal transducer and activator of transcription 5 (pSTAT5), which in turn regulates the expression of GATA-binding protein 3 (GATA3), which is required for ILC2 development.
Munneke et al. [51]	2014	Graft versus host disease	In 51 patient affected by leukemia, ILC2 reduced the susceptibility to therapy-induced mucositis and acute graft versus host disease.
Bruce et al. [52]	2021	Graft versus host disease	A mouse model highlighted that third-party ILCs significantly improve the survival of mice after allo-hematopoietic stem cell transplantation (HSCT) when given at the time of transplantation.
Van der Ploeg et al. [53]	2020	Mastocytosis	A total of 21 patients with systemic mastocytosis were compared to 18 healthy controls and it was found that patients with skin involvement and pruritic symptoms had higher ILC2 levels, independent of serum tryptase levels.

**Table 2:** The main findings about the role of ILC2s in allergic cutaneous inflammation.

Authors	Year	Topic	Study Characteristics
Roediger et al. [54]	2013	Cutaneous type 2 inflammation	Two functional states of dermal group 2 innate lymphoid cells (ILC2s): steady-state ILC2s with immuno-regulatory action, and stimulated ILC2s with a pro-inflammatory activity.
Nakatani-Kusakabe et al. [55]	2021	Cutaneous type 2 inflammation	- Thymic stromal lymphopoietin (TSLP) activates cutaneous ILC2; - Interleukin (IL)-33 favors the migration of MHCII+-ILC2s to lymph nodes, where ILC2s take part in adaptive immune response.
Leyva-Castillo et al. [56]	2020	Cutaneous type 2 inflammation	- Crosstalk between keratinocytes and ILC2s; - IL-25 produced by keratinocytes stimulates cutaneous ILC2s, consequently activating T helper cells, increasing the production of IL-13 and T-cell attracting chemokines and promoting epidermal hyperplasia.
Ricardo-Gonzalez et al. [57]	2018	Cutaneous type 2 inflammation	Subpopulations of ILC2s expressing receptors for IL-18 could be partly responsible for the most severe forms of allergic skin inflammation and increased levels of IL-18 that correlate with severity of atopic dermatitis.
Hsu et al. [59]	2020	Cutaneous type 2 inflammation	- Crosstalk between basophils and ILC2s; - Production of IL-4 by a direct mechanism; - Chemotactic action of IL-4 toward ILC2s increases their responsiveness to epithelium-derived cytokines, which in turn, regulate cytokine production by basophils; - Prostaglandin 2 (PDG2)-CRTH2 binding and IL-13 produced by ILC2s could reduce mast cell activation.
Kim et al. [58]	2014	Cutaneous type 2 inflammation	- Crosstalk between basophils and ILC2s; - Production of IL-4 by a direct mechanism; - Chemotactic action of IL-4 toward ILC2s increases their responsiveness to epithelium-derived cytokines, which in turn, regulate cytokine production by basophils; - Involvement of leukotriens.
Malhotra et al. [60]	2018	Cutaneous type 2 inflammation	Certain polymorphisms in the gene encoding for RORalpha may underlie the genesis of Th2/ILC2-mediated skin inflammatory diseases.
Rafei-Shamsabadi et al. [61]	2018	Allergic contact dermatitis	ILC2 activation would be critical for type 2 skin inflammation in allergic contact dermatitis, and its lack would promote a type 1 immune response.
Shane et al. [62]	2019	Skin allergic disease to didecyldimethylammonium chloride	ILC2s could be the potential mediator of immunologic reaction skin allergic diseases with an underlying mixed IgE-mediated and T-cell-mediated immunologic mechanism.
Tsai et al. [63]	2019	Drug Rash with Eosinophilia and Systemic Symptoms	- The stimulation of ILC2s by IL-33 is responsible for skin manifestations, eosinophilic inflammation, and poor response to corticosteroids; - Contribution to reparative mechanisms, skin remodeling, and fibrosis through the expression of ST2 and the rapid response of ILC2s to IL-33 stimulation, releasing epidermal growth factors and activating eosinophils.
Numazaki et al. [64]	2019	Cutaneous inflammation	Monoclonal anti-TSLP antibody reduces ILC2 activation and resolves the resistance that ILC2s develop toward steroids.

**Table 3:** The main finding about the role of ILC2s in atopic dermatitis.

Authors	Year	Study Characteristics
Nishikawa et al. [67]	2021	A mouse model using transgenic binary mice that are constitutively deficient in conventional dendritic cells (cDCs) demonstrated that congenital cDCs deficiency not only exacerbated atopic dermatitis (AD)-related inflammation but also resulted in immune alterations as the composition and function of the cells increased granulocytes and group 2 innate lymphoid cells (ILC2s).
Früh et al. [66]	2020	In a canine model, levated frequencies and numbers of T-helper 2 cells but not ILC2s in peripheral blood were associated with chronic canine atopy.
Imai et al. [78]	2019	In a mouse model, ILC2 cells were depleted in IL33tg mice via bone marrow transplantation; in these mice, the development of inflammation was almost completely suppressed, demonstrating the central role of ILC2s in immune processes.
Salimi et al. [70]	2013	The binding of E-cadherin to human ILC2s dramatically inhibited interleukin (IL)-5 and IL-13 release. In the absence of E-cadherin, which is characteristic of filaggrin insufficiency and feature of AD, ILC2s resulted in increased production of type 2 cytokines.
Salimi et al. [74]	2015	The expression of NKp30 ILC2s ex vivo and on cultured ILC2s and the interaction with its ligand B7-H6 induced the production of type 2 cytokines in AD.
Baek et al. [85]	2017	In a mouse model, immune tolerance was induced by oral administration of ovalbumin with subsequent epicutaneous sensitization by repeated application of ovalbumin. The induction of oral tolerance resulted in a reduction in the inflammatory response through the inhibition of ILC2 levels.
Perrone Sibilia et al. [84]	2019	Chronic infection with *Toxoplasma gondii* appeared to prevent the development of atopic dermatitis through a reduced susceptibility that appeared to result from changes in the type II innate immune response, with reductions in IL-4, IL-5, and ILC2s.
Lee et al. [82]	2021	Celastrol decreased the levels of thymic stromal lymphopoietin (TSLP), ILC2s, and T-helper 2-cytokines in AD lesions of house dust mite-stimulated NC/Nga mice.
Mashiko et al. [68]	2017	AD lesional skin, but not psoriasis skin, was infiltrated by increased numbers of basophils, which produced IL-4,T-helper 2 cells and ILC2s.
Schwartz et al. [79]	2019	ILC2 deficiency did not improve AD in Flgft/ft mice, which was also independent of IL-4, IL-5, IL-9, IL-13, IL-17A, and IL-22, but required the signaling of IL-1β and IL-1R1, which therefore, also play a fundamental role in the development of inflammation.
Natsume et al. [83]	2020	A study comparing the anti-itch effect of xanthophyll with tacrolimus observed that the former significantly reduced symptoms compared to the latter through the presence of ILCreg suppressing the immune response.
Hardman et al. [73]	2017	In a human AD-based model, TSLP-induced ILC2s expressed Cluster of Differentiation 1a (CD1a), which, in turn, activated TH lymphocytes. In addition, ILC2s presented endogenous lipid antigens to CD1a-reactive T cells.

**Table 4:** The main finding about the potential therapeutic role of ILC2s in melanoma, AD and cutaneous type 2 inflammation.

Authors	Target	Disease	Immunological Mechanism
Okuyama et al. [38]	IL-33/ST2 interaction	melanoma	Group 2 innate lymphoid cells (ILC2s), once stimulated by interleukin (IL)-33 after binding with their ST2 receptor, play a tumor role activating CD8+ T-cell expression and infiltration into the tumor and inducing an unfavorable tumor environment.
Jacquelot et al. [36]	PD1	melanoma	Inhibition of programmed cell death protein (PD-1) results in increased total ILC2s number. High ILC2s infiltration in human melanoma is associated with a good clinical prognosis.
Wagner et al. [37]	Lactic acid	melanoma	Acid lactic inhibits ILC2 proliferation and cytokine production, exerting immunosuppressive activity against ILC2s. Conversely. in knockdown lactate dehydrogenase A B16F10 melanomas (LDHAlow), the number of intratumoral ILC2s increased proportionally.
Ricardo-Gonzalez et al. [57]	IL-18	Cutaneous type 2 inflammation	- Subpopulations of ILC2-expressing receptors for IL-18 are responsible for the most severe forms of allergic skin inflammation; - Increased levels of IL-18 correlate with the severity of dermatitis; - IL-18 and IL-33 action on ILC2s would appear to be closely related.
Numazaki et al. [64]	TSLP	Cutaneous inflammation	In vitro study demonstrated that monoclonal anti-thymic stromal lymphopoietin (TSLP) receptor antibodies managed to reduce both type 2 and non-type 2 inflammation.
Salimi et al. [74]	NKp30	AD	Cultured ILC2s subset expressing NKp30, after interaction with its activator ligand B7-H6, rapidly produce type 2 cytokines.

## Data Availability

Not applicable.
